# Kutane Ulzerationen bei Dermatomyositis: Fallberichte mit positivem Anti-NXP2/Anti-TIF1-gamma-Antikörper-Status

**DOI:** 10.1007/s00105-024-05462-0

**Published:** 2025-01-16

**Authors:** Caroline Wald, Jan-Christoph Simon, Regina Treudler

**Affiliations:** 1 Klinik für Dermatologie, Allergologie und Venerologie, Universitätsklinikum Leipzig, Philipp-Rosenthal-Str. 23, 04103 Leipzig, Deutschland; 2 Institute for Allergology, Charité – Universitätsmedizin Berlin, Corporate Member of Freie Universität Berlin and Humboldt-Universität zu Berlin, Berlin, Deutschland

**Keywords:** Dermatomyositis, Ulzeration, Anti-MDA5-Antikörper, Immunsuppressive Therapie, Topische Kortikosteroidtherapie, Dermatomyositis, Ulceration, Anti-MDA5 antibody, Immunosuppression therapy, Topical corticosteroid therapy

## Abstract

Eine 33-jährige Patientin mit Anti-NXP2(„nuclear matrix protein 2“)-positiver und eine 67-jährige Patientin mit TIF1-gamma(„transcription intermediary factor 1 gamma“)-positiver Dermatomyositis entwickelten unter Systemtherapie mit Azathioprin bzw. Low-dose-Methylprednisolon und zyklischer Verabreichung von intravenösen Immunglobulinen (IVIGs) schmerzhafte Ulzerationen im Gesichts- und Körperstammbereich bzw. an den Händen. In der laborchemischen Diagnostik blieb bei beiden Patientinnen der Anti-MDA5(„anti-melanoma differentiation-associated protein 5“)-Antikörperstatus negativ und die Kreatinkinase (CK) normwertig, die histopathologischen Untersuchungen erbrachten ein unspezifisches Ergebnis. Eine intensive topische Kortikosteroidtherapie der Klasse 4 und Fortführung der immunsuppressiven bzw. immunmodulierenden Therapie führten zur Abheilung der ulzerativen Hautveränderungen. Dieser Bericht soll für das seltene Auftreten kutaner Ulzerationen bei einer Anti-NXP2- bzw. Anti-TIF1-gamma-positiven Dermatomyositis sensibilisieren, da Ulzerationen mit einem schweren Krankheitsverlauf assoziiert sein können. In unseren beiden Patientenfällen handelt es sich um eine ausschließlich dermale Ausprägung ohne sonstige Komplikationen.

## Abstract (en)

Painful ulcerations developed in a 33-year-old woman with anti-NXP-2-positive dermatomyositis in the facial and trunk areas and a 67-year-old woman with TIF1-gamma-positive dermatomyositis on the hands, while undergoing systemic therapy with azathioprine or low-dose methylprednisolone and cyclic administration of intravenous immunoglobulins (IVIG), respectively. In the laboratory workup, the anti-MDA‑5 antibody status remained negative and the creatine kinase (CK) normal in both patients, while histopathological examinations were nonspecific. Intensive topical class 4 corticosteroid therapy and continuation of the immunosuppressive or immunomodulating therapy led to healing of the ulcerative skin lesions. This report aims to raise awareness of the rare occurrence of cutaneous ulceration in anti-NXP-2- or anti-TIF1-gamma-positive dermatomyositis, as ulcerations may be associated with a severe disease course. In both of our patient cases, it is exclusively a dermal manifestation without other complications.

Die Dermatomyositis ist eine systemische Autoimmunerkrankung, die die Muskeln und die Haut betreffen kann [1]. Die kutanen Manifestationen sind heterogen und umfassen typischerweise Gottron-Papeln, Gottron-Zeichen und ein heliotropes Erythem [2]. Fachberichten zufolge zeigen 3–19 % der Betroffenen kutane Ulzerationen [1]. Ulzerationen spiegeln vermutlich eine Vaskulopathie wider, bedingt durch Hypoxie und Ischämie des betroffenen Gewebes [3]. Weitere potenzielle Auslösefaktoren sind eine Vaskulitis, übermäßige Inflammation an der dermoepidermalen Junktionszone oder Exkoriation [1].

In der Literatur wird eine Assoziation zwischen kutanen Ulzera und Anti-Ro52- sowie Anti-MDA5(„anti-melanoma differentiation-associated protein 5“)-Antikörpern (AK) beschrieben, die mit einem erhöhten Risiko für die Entwicklung einer interstitiellen Lungenerkrankung vergesellschaftet sind. Zwischen den übrigen Dermatomyositis-AK (TIF1-gamma [„transcription intermediary factor 1 gamma“], Mi2, NXP2 [„nuclear matrix protein 2“], Jo‑1, SAE1) und Ulzera wurde kein statistisch signifikanter Zusammenhang gefunden [1].

Wir berichten über Hautulzerationen bei zwei Patientinnen ohne Anti-MDA5-Antikörpernachweis.

## Kasuistik 1

### Anamnese und klinischer Befund

**Abb. 1:**
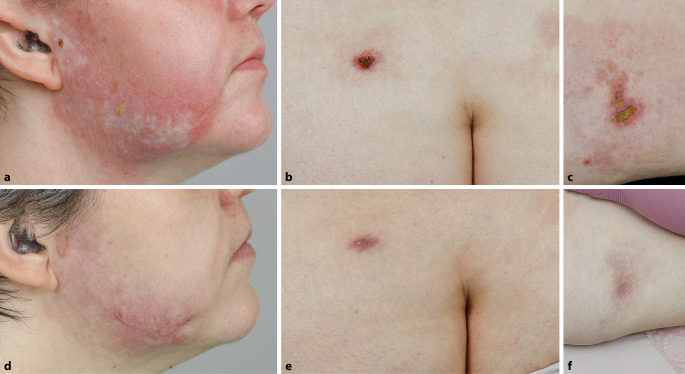
**a–c** Ulzerativer Befund der Patientin aus Kasuistik 1. **d–f** Abgeheilte Läsionen nach ca. 3 Monaten. **a**, **d** Rechte Gesichtshälfte, **b**, **e** unterer Rücken, **c**, **f** Abdomen

Eine 33-jährige Patientin zeigte livide Erytheme periorbital, am Kieferwinkel und am Dekolleté sowie Muskelschmerzen und Kraftminderung der proximalen Extremitäten. Histologisch zeigte sich ein lymphozytäres Entzündungszellinfiltrat mit vaskulärer Degeneration der basalen Keratinozyten, elektromyographisch waren pathologische myogene Veränderungen nachweisbar. Antinukleäre Antikörper (ANA) waren negativ und die Kreatinkinase (CK) war mit 21,5 µkat/l (Grenzwert 0,43–2,34 µkat/l) erhöht. Bei Nachweis von Antikörpern (AK) gegen das „nuclear matrix protein 2“ (NXP2) wurde die Diagnose einer Dermatomyositis gestellt. Die Tumordiagnostik verlief unauffällig. Die Patientin erhielt mehrfach Methylprednisolon-Stoßtherapien (3 Tage jeweils 500 mg) sowie 5 Zyklen intravenöse Immunglobuline (IVIG) mit 2 g/kg Körpergewicht (KG). Eine Dauertherapie wurde mit Azathioprin (1 g/kgKG) eingeleitet. Acht Monate nach Diagnosestellung zeigten sich erstmals am rechten Unterkiefer, abdominell, am Gesäß, Rücken und an beiden proximalen Oberschenkeln gelb-krustig belegte, schmerzhafte Ulzerationen (Abb. 1a–c).

### Diagnostik

Die Anti-NXP2-AK waren erneut grenzwertig positiv, Anti-MDA5-Antikörper waren nicht nachweisbar. Die CK war normwertig. Histologisch zeigte sich eine Akanthose mit Gewebedefekt und superfiziellem, perivaskulärem lymphozytärem Entzündungszellinfiltrat.

### Therapie und Verlauf

Bei Lymphopenie wurde Azathioprin auf 50 mg (0,5 mg/kgKG) reduziert und Hydroxychloroquin (2 g/kgKG) eingeleitet. In Kombination mit antiseptischen und Glukokortikosteroid-haltigen Externa der Klasse 4 und Fortführung der IVIG-Therapie zeigten sich die Ulzerationen und das gesamte Krankheitsbild regredient (Abb. 1d–f). In der 1‑jährigen Krankheitsdauer wurden keine Hinweise auf eine kardiale oder pulmonale Beteiligung der Erkrankung beobachtet.

## Kasuistik 2

### Anamnese und klinischer Befund

Eine 67-jährige Patientin zeigte Erytheme im Gesicht, an den Händen und am Dekolleté sowie Muskelkraftverlust der proximalen Extremitäten. Es gelang histologisch und laborchemisch der Nachweis einer Anti-TIF1-gamma(„transcription intermediary factor 1 gamma“)-positiven Dermatomyositis. ANAs waren mit 1:640 und die CK mit 21,6 µkat/l erhöht. Eine Systemtherapie mit Azathioprin (ca. 1,5 mg/kgKG) wurde nach einem Jahr aufgrund einer Lymphopenie beendet. Die Patientin erhielt mehrere hoch-dosierte Methylprednisolon-Stoßtherapien über 3 Tage bis maximal 250 mg/Tag, später Zyklen von IVIG (2 g/kgKG) unter einer Erhaltungsdosis mit 4 mg Methylprednisolon 1‑mal täglich. Zwei Jahre nach Diagnosestellung wurde ein Bronchialkarzinom diagnostiziert und zeitnah reseziert.

**Abb. 2:**
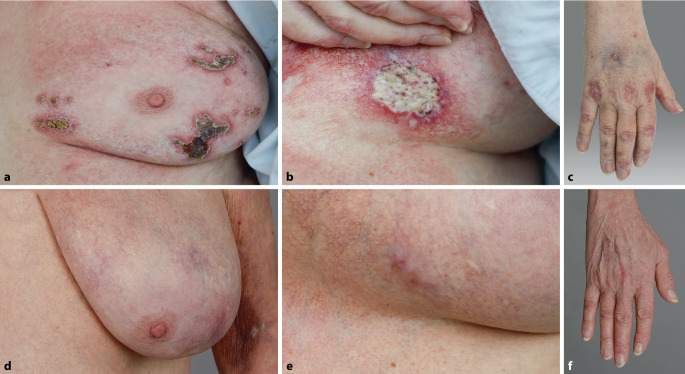
**a–c** Ulzerativer Befund der Patientin aus Kasuistik 2. **d–f** Abgeheilte Läsionen nach 1,5 Jahren. **a**, **d** Linke Mamma, **b**, **e** submammär links, **c**, **f** rechte Hand

Vier Monate nach Tumorresektion traten erstmals Ulzerationen mammär und inguinal auf, später zervikal, an der rechten Hand, genital und anal (Abb. 2a–c) bei normwertiger CK.

### Diagnostik

Histologisch wurde eine Ulzeration mit reaktiver Epithelhyperplasie gesichert. Der Anti-MDA5-Antikörperstatus blieb negativ.

### Therapie und Verlauf

Zusätzlich zur topischen antiseptischen Behandlung wurden mehrere Zyklen Iloprost intravenös, im späteren Verlauf Bosentan verabreicht. Die Gabe von IVIG unter niedrig-dosierter Erhaltungsmedikation mit Methylprednisolon (32 mg, ausschleichende Reduktion auf 16 mg) wurde fortgesetzt. Eine topische Therapie erfolgte mit Glukokortikosteroid-haltigen Externa der Klasse 4. Die Patientin erkrankte im Juli 2022 schwer an SARS-CoV‑2 („severe acute respiratory syndrome coronavirus type 2“) mit der Notwendigkeit einer intensivmedizinischen Behandlung. Sechs Monate später stellte sie sich erneut mit vollständig abgeheilten Ulzerationen vor (Abb. 2d–f). Während der 4‑jährigen Krankheitsdauer gab es keinen Hinweis auf eine kardiale oder pulmonale Beteiligung der Dermatomyositis.

## Diskussion

Kutane Ulzerationen bei Dermatomyositis sind typischerweise schmerzhaft und beeinträchtigen die Lebensqualität. Typische Lokalisationen beinhalten die Streckseiten über den Gelenken (Finger, Ellbogen und Knie), die seitlichen Nagelfalze oder die digitale Pulpa, die Brust und die Ohrhelix. Ulzerationen werden in der Literatur bei Anti-Ro52- und Anti-MDA5-AK beschrieben, wobei Letztere häufig mit einer rasch fortschreitenden interstitiellen Lungenerkrankung assoziiert sind [1].

Ulzerationen bei Anti-NXP2- und Anti-TIF1-gamma-AK sind – wie bei unseren Patientinnen – phänotypisch ungewöhnlich und werden kasuistisch berichtet [4, 5].

Wang et al. [6] beschrieben eine 2‑jährige Patientin mit Anti-NXP2-AK-positiver juveniler Dermatomyositis und Hautulzerationen. Rossells et al. [7] berichteten über rasch progrediente Ulzerationen bei einem Patienten mit malignomassoziierter Dermatomyositis, der positive Anti-TIF1-gamma/alpha- und Anti-U1-RNP („U1-Ribonukleoprotein“)-AK aufwies und im Verlauf an einer Infektion verstarb.

Unabhängig vom Anti-MDA5-Antikörperstatus wurde das Vorhandensein von Ulzerationen bei Dermatomyositis mit einem schwereren Krankheitsverlauf assoziiert [8]. So können Komplikationen wie Gangrän oder Osteomyelitis auftreten [1]. Zudem besteht eine ungünstige Prognose für die Krankheitskontrolle aufgrund einer erhöhten Resistenz gegenüber immunsuppressiven Therapien [1]. In den von uns präsentierten Kasuistiken lag eine ausschließlich dermale Manifestation vor bei laborchemisch normwertiger CK ohne Auftreten von weiteren Komplikationen.

Bisher existiert keine Standardtherapie für diese Ulzerationen. Immunsuppressiva wie Azathioprin, Cyclophosphamid und Cyclosporin als auch immunmodulierende Therapien wie Hochdosissteroid oder IVIG können zur Besserung beitragen [8]. Vasodilatatoren (Phosphodiesteraseinhibitoren, Kalziumkanalantagonisten), Endothelin-Rezeptor-Inhibitoren oder rheologisch fördernde Medikamente (Pentoxifylline) können ebenfalls hilfreich sein [9]. Bei unseren Patientinnen konnte unter Fortführung der immunsuppressiven sowie intensivierten kortikosteroidhaltigen topischen Therapie (Klasse 4) eine Abheilung der Ulzerationen beobachtet werden.

## Fazit für die Praxis

- Kutane Ulzerationen können, auch wenn phänotypisch ungewöhnlich, bei NXP-2(„nuclear matrix protein 2“)- bzw. Anti-TIF1-gamma(„transcription intermediary factor 1 gamma“)-positiver Dermatomyositis auftreten.
- Unabhängig vom Anti-MDA5(„anti-melanoma differentiation-associated protein 5“)-Antikörperstatus können Ulzerationen mit einem schwereren Krankheitsverlauf verbunden sein.
- Eine Abheilung kann unter Fortführung der immunsuppressiven sowie intensivierten kortikosteroidhaltigen topischen Therapie erreicht werden.
